# Pygo2+ T cells possess immunosuppressive features and inferior immunotherapeutic response in gastric cancer

**DOI:** 10.3389/fimmu.2025.1596434

**Published:** 2025-07-23

**Authors:** Weilong Chang, Huifang Yan, Yawei Zhang, Zibo Sang, Bei Bu, Rui Deng, Kaibo Li, Jiajing Li, Yang Fu, Jinyuan Cui

**Affiliations:** ^1^ Department of Gastrointestinal Surgery, The First Affiliated Hospital of Zhengzhou University, Zhengzhou, Henan, China; ^2^ Department of Radiotherapy, The Second Affiliated Hospital of Zhengzhou University, Zhengzhou, Henan, China; ^3^ Department of Clinical Medicine, The First Clinical Medical College of Zhengzhou University, Zhengzhou, Henan, China; ^4^ Reproductive Medical Center, The First Affiliated Hospital of Zhengzhou University, Zhengzhou, Henan, China; ^5^ Department of Pathology, The First Affiliated Hospital of Zhengzhou University, Zhengzhou, Henan, China

**Keywords:** gastric cancer, immunotherapeutic response, Pygo2, single-cell sequencing, immune microenvironment

## Abstract

**Background:**

Gastric cancer (GC) poses a significant threat to human health. Despite considerable advancements in immunotherapy for GC, the effectiveness of current immunotherapeutic targets remains constrained by the heterogeneity of the tumor microenvironment and mechanisms of immune evasion. Consequently, the identification of novel immunotherapy targets has emerged as a critical area of research. This study investigates the potential of Pygo2 as a target for immunotherapy in GC.

**Methods:**

The expression and cell localization of Pygo2 in GC tissues were characterized by single cell sequencing, flow cytometry and mIHC. The relationship among Pygo2 expression and prognosis, immune microenvironment and immunotherapy effect was studied in 282 gastric cancer patients.

**Results:**

The findings indicate a significant upregulation of Pygo2 expression in GC tissues, particularly within tumor cells and T cells. Pygo2 expression in T cells is not only correlated with the advanced T stage and N stage but also inversely associated with patient survival. Additionally, overexpression of T cell Pygo2 resulted in a significant increase in TCF7, which suggested Pygo2^+^ T cells might represent a subset of exhausted T cells. The study also demonstrated that the density of Pygo2^+^ CD8^+^ T cells is negatively correlated with the efficacy of immunotherapy.

**Conclusion:**

Tumor-infiltrating Pygo2^+^ T cells could be applied as a clinical prognosticator and a predictive biomarker for immunotherapy responsiveness to GC. These findings offer new therapeutic targets for the treatment of GC and provide fresh insights into cancer treatment strategies.

## 1 Introduction

Gastric cancer (GC) represents a malignant neoplasm that poses a substantial risk to global health. Current epidemiological data reveal an annual incidence of approximately 1 million new GC cases worldwide, with nearly 800,000 fatalities (1). Recent advancements in the treatment modalities for GC have demonstrated significant progress, particularly in the areas of precision medicine, immunotherapy, and targeted therapy (2–4). Immunotherapy is a promising strategy in the management of GC, owing to its ability to specifically target and disrupt GC cells to achieve therapeutic goals. Immunotherapy is one of the effective approaches for the treatment of GC, as it is capable of specifically targeting tumor sites and activating immune cells to disrupt or eliminate GC cells, thereby fulfilling therapeutic objectives (5, 6). The main immunotherapy targets for GC identified so far include PD-1/PD-L1 and CTLA-4, etc (7–9). However, some factors, such as the diversity of the immune microenvironment of GC and immune escape mechanisms, limit the effectiveness of existing immunotherapy targets, resulting in poor therapeutic effects (10, 11). Therefore, the search of new immunotherapeutic targets of GC becomes a key area of research, especially the study of new targets within the immune system, which has great potential to enhance the treatment of this malignancy.

Pygopus homolog 2 (Pygo2) is a protein characterized by PHD and Bromo domains and it is classified as a member of the Pygo family. The PHD domain is able to interact with histone methyltransferases and acetylases, and the PHD domain is primarily associated with transcriptional cofactors, including histone 3 and Bcl/9l (12, 13). These domains are critical for the modulation of chromatin structure and the activation of Wnt target genes (12, 14). Pygo2 plays a critical role in the development of specific tumors, with the Wnt/β-catenin signaling pathway potentially acting as a key regulatory. Some studies indicate that Pygo2 enhances Wnt/β-catenin signaling by inhibiting the expression of antagonists of Wnt signaling (15, 16). Additionally, research reveals that Pygo2 and β-catenin jointly regulate the expression of miR-29 family members, which contributes to the dedifferentiation of mammary epithelial tumor cells (17). In another study, the lack of the Pygo2 gene is associated with activation and infiltration of cytotoxic T lymphocytes (CTLs). This observation suggests that the effects of Pygo2 on tumors are mediated through T cells (13). However, the specific mechanism of the interaction between Pygo2 and T cells remains to be elucidated. In addition to prostate and breast cancer, Pygo2 also significantly promotes the occurrence and development of other malignancies, including esophageal cancer, colon cancer, and liver cancer (18–20). Unfortunately, the functional role and mechanism of action of Pygo2 in GC have not been fully studied.

This research seeks to examine the function and underlying mechanisms of Pygo2 in the progression of GC. By employing single-cell sequencing analysis and multiplex immunohistochemistry techniques (mIHC), the study clarifies the expression patterns and localization of Pygo2 within GC tissues. In addition, it also investigates the relationship among Pygo2 expression in immune cells, patient prognosis, immune microenvironment status, and immunotherapy efficacy. The investigation introduces novel therapeutic targets for future interventions in GC, aiming to enhance both treatment specificity and efficacy. Furthermore, the article offers preliminary insights into the potential interactions between Pygo2 and T cells, thereby providing new avenues for future cancer treatment strategies.

## 2 Materials and methods

### 2.1 Patient samples

This study involved the selection of 282 GC patient samples from Shanghai OUTDO Biotech Co. (https://superchip.com.cn/), and the process was approved by the ethics committee of Shanghai OUTDO Biotech Co (SHYJS-CP-1910016). The staging process followed the guidelines outlined in the 7th edition of the TNM staging guidelines by the American Joint Committee on Cancer (AJCC). None of the patients had undergone chemotherapy or radiotherapy prior to tumor resection. 30 cases of GC and corresponding normal gastric tissues were obtained from radical gastrectomy specimens from the Department of Gastrointestinal Surgery at the First Affiliated Hospital of Zhengzhou University. Additionally, 10 GC patients who underwent neoadjuvant immunotherapy were identified from the Department of Gastrointestinal Surgery at the First Affiliated Hospital of Zhengzhou University (**Supplementary Table S1**). Fresh GC tumors and adjacent normal liver tissues were collected for subsequent research and analysis. The adjacent normal tissue was required to be at least 2 cm away from the corresponding tumor tissue. The Institutional Review Board of the First Affiliated Hospital of Zhengzhou University approved using the tumor specimens in this study.

### 2.2 Immunohistochemistry

The patient samples should be fixed in 4% paraformaldehyde in 0.1 M phosphate-buffered saline, followed by embedding in paraffin and sectioning into 4 μm slices. Immunostaining should be conducted in accordance with the following specific protocol: incubate the sections overnight at 4°C with the monoclonal antibody Pygo2 (1:5000; Abcam, #ab316318), PD-1 (1:100; CST, #86163), TIGIT (1:200; Abcam, #ab243903), and TIM-3 (1:100, Abcam, #ab242080). A second incubation was then performed for one hour at room temperature with an HRP-conjugated secondary antibody before using the DAB Detection Kit (Polymer) (Gene Tech, #GK600510) to perform the peroxidase reaction. Finally, the sections should be examined using a microscope (Olympus Japan CX33-LV2000), and the results should be recorded. Two pathologists blinded to clinical data separately assessed Pygo2 expression by tumor cells and T cells. Positive cell staining density was determined based on the stained cells observed per field of view (cells/mm^2^) with the Aipathwell software by Servicebio (China).

### 2.3 Multiplex immunohistochemistry

According to the manufacturer’s instructions, multiplex staining was performed using the PDOne six colors kit (PANOVUE, #10234100050). First, the GC tissue samples are incubated with the primary antibody at 37°C for 1 h. Then, the sections are slowly rinsed three times in TBST buffer, with each rinse lasting 5 min. Then, the sections are slowly rinsed three times in TBST buffer, with each rinse lasting 5 min. Next, the sections are incubated with HRP labeled secondary antibodies at 37°C for 30 min. Finally, they are incubated with PANO TSA staining buffer at room temperature for 15 min. The steps of incubating with the primary antibody, secondary antibody, and PANO TSA staining are repeated until the marker shows color. The primary antibodies used include Pygo2 (1:5000; Abcam, #ab316318), CD8 (1:500; CST, #85336), CD4 (1:600; Abcam, #ab133616), and Pan-CK (1:500; Abcam, #ab215838). All slides are stained with DAPI at room temperature for 5 min and imaged using a multifunctional spectral imaging system (PerkinElmer). The scanned images are imported into HALO software for analysis. Based on a nuclear segmentation algorithm, the nuclei in all samples are identified and the cell count is determined. Using the CytoNuclear algorithm in HALO, the quantity of various immune cells is detected based on nuclear characteristics.

### 2.4 Flow cytometry

Freshly isolated GC tissues were cut into small pieces and digested with Collagenase IV and DNase I at 37°C on a shaking bed for 30 minutes to achieve complete tissue digestion. The obtained cells were lysed with Lysis buffer and kept on ice. After erythrocyte lysis, the samples were then incubated with a human BD Fc blocker and stained with the LIVE/DEAD Cell Imaging Kit (Invitrogen) in the dark at room temperature for 30 minutes. A series of antibodies, including CD45, CD4, CD8, and Pygo2, were co-incubated with the cells in the stain buffer for 30 minutes. The stained cells were washed and resuspended in the staining buffer before being separated in a Cytoflex LX flow cytometer and analyzed using FlowJo software (version 10).

### 2.5 T cell isolation and transfection

We extracted peripheral blood T cells from the same gastric cancer patient using the methods reported in previous studies (11, 21, 22). In brief, we isolated peripheral blood mononuclear cells by density gradient centrifugation (Stemcell Technologies, Vancouver, BC, Canada). Then anti-CD3 (100 ng/ml) antibody and IL-2 (10 ng/ml) were added to stimulate peripheral blood mononuclear cells for 5 to 7 days. The T cell population was then extracted and amplified using the ImmunoCult Human CD3/CD28 T Cell Activator (25 µl/mL, Stemcell Technologies). We constructed a Pygo2-hTLv lentivirus suitable for T cell transfection, and constructed Pygo2-overexpressing T cells according to the instructions of the reagent manufacturer (Shanghai Hanbio Co, Ltd).

### 2.6 Western blot

Total protein was extracted using RIPA lysis buffer (Thermo Fisher Scientific), and the protein concentration was determined using the BCA method. For each well, 20 mg of denatured total protein was loaded onto a 10% SDS-PAGE gel for electrophoresis, and then transferred to a PVDF membrane. The membrane was subsequently blocked with a TBST solution containing 5% skimmed milk for one hour. Primary antibodies were properly diluted and incubated with the membrane overnight at 4°C. Immunodetection was performed applying anti-Pygo2 (Abcam, #ab316318), anti-β-catenin antibody (CST, #8480), anti-TCF7 antibody (CST, #2203), and anti-Myc-antibody (CST, #5605). Following three washes with TBST, the membrane was incubated with corresponding secondary antibodies for one hour at room temperature. All protein blots were captured by iBright imaging system (Invitrogen).

### 2.7 Acquisition of data from public database

Single-cell sequencing data of human GC from GSE163558 was obtained from the Gene Expression Omnibus (GEO) database. The bulk RNA sequencing data, survival data, and clinicopathologic data from four separate groups of GC patients (TCGA-STAD and GSE27342) were obtained from The Cancer Genome Atlas (TCGA) database, and GEO database, respectively.

### 2.8 Single-cell sequencing analysis and bulk RNA sequencing analysis

Single-cell sequencing data from GSE163558 was conducted a sequence of analysis to identified Pygo2+ cells. The raw gene expression matrix was converted into a Seurat object via Seurat R package (V5.0.1). Cells with >6000 or <200 genes or >20% mitochondrial genes were discarded. Potential doublets were removed using the DoubletFinder R package (V2.0.4). Data were normalized, scaled, and subjected to principal component analysis. Principal components analysis (PCA) was performed using the 2000 highly variable genes identified by the Find Variable Features function in Seurat. Distinct clusters of cells were identified using the first 30 PCA components for graph-based clustering with a resolution of 0.8. The Unified Manifold Approximation and Projection (UMAP) method visualized single-cell clusters. We estimated the differentially expressed genes of each cluster using the Find All Markers module, and genes expressed in more than 25% of the cells were selected. The DEGs expressed only in Pygo2+ T cells were considered the characteristic gene signature of Pygo2+ T cells. The cell-cell interaction network was analyzed and visualized using the CellChat R package (version 1.6.1). Single-sample gene set enrichment analysis (ssGSEA) was implemented to estimate the infiltration of Pygo2+ T cells of each sample in TCGA cohort.

### 2.9 Statistical analyses

The statistical analysis of RNA-seq data, single-cell sequencing data, and mIHC data was performed using GraphPad Prism version 8.0 and SPSS version 23.0 software and R software. The results are expressed as the mean ± standard deviation (S.D.), and group differences were assessed using the chi-square test. Differences in continuous variables between groups were analyzed using the t-test. The log-rank test was used to compare survival curves drawn using the Kaplan–Meier curves. A P value of less than 0.05 was considered statistically significant, with * representing P < 0.05, ** representing P < 0.01, and *** representing P < 0.001. All reported P values are based on two-tailed test results.

## 3 Results

### 3.1 Pygo2 expression was abnormally high in GC

**Figure 1:**
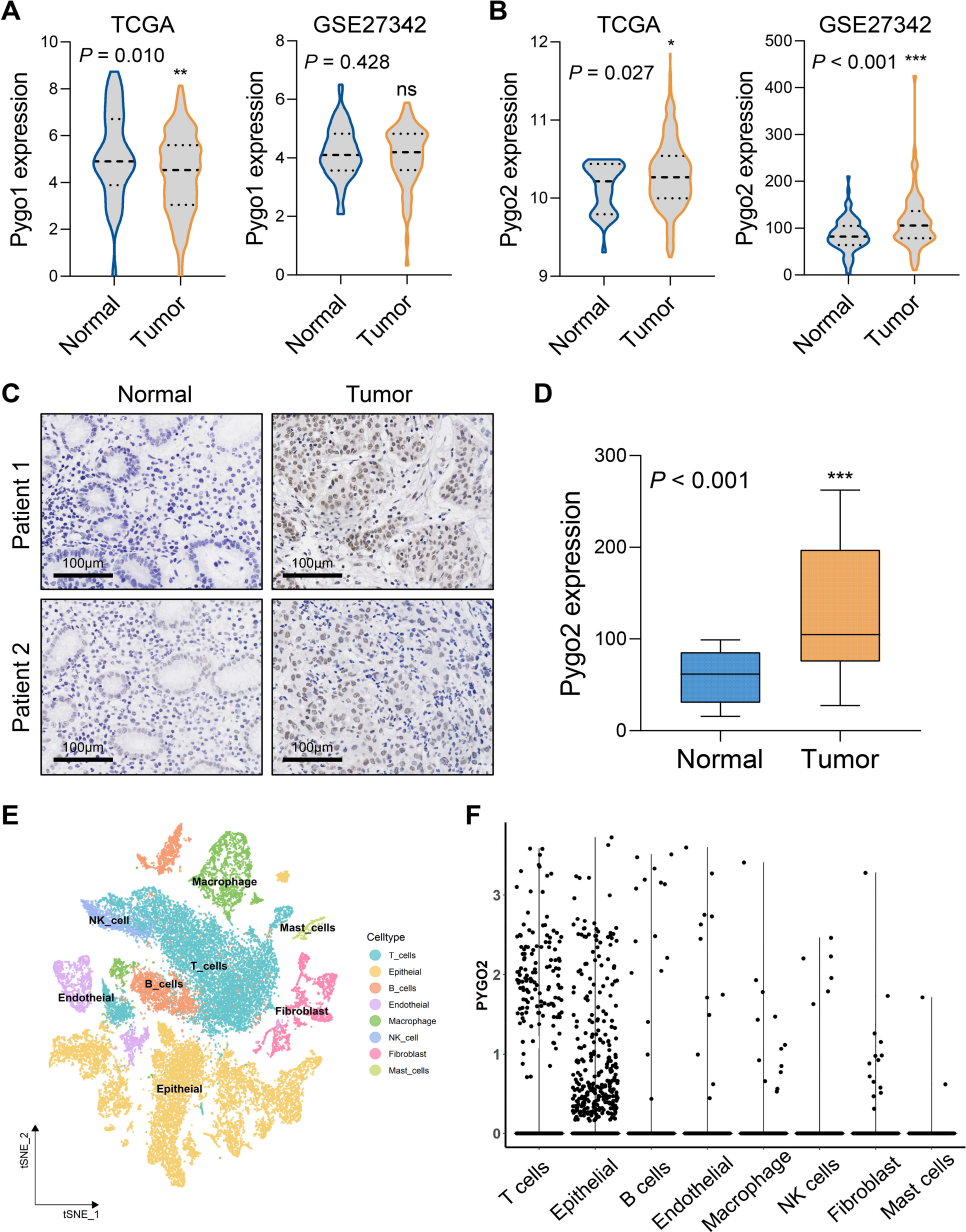
Pygo2 expression was abnormally high in GC. **(A)** The mRNA expression difference of Pygo1 in GC tissues and normal tissues was analyzed by TCGA and GEO databases. **(B)** The mRNA expression difference of Pygo2 in GC tissues and normal tissues was analyzed by TCGA and GEO databases. **(C)** Representative images of IHC staining of Pygo2 showing the differential expression between GC and corresponding normal tissues. **(D)** The box plot outlining the expression level of Pygo2 in 30 pairs of GC and normal tissues. **(E)** UMAP plot representation of 8 unique cell clusters color coded by their corresponding immune cell subtype. **(F)** Pygo2 expression levels in different cell types were obtained from single cell sequencing data.

The Pygo family primarily consists of Pygo1 and Pygo2. An analysis of mRNA expression differences in the Pygo family between GC tissues and normal tissues was conducted using data from the TCGA and GSE27342 databases. The results indicated that there was no significant difference in the transcription levels of Pygo1 between the two tissue types (**Figure 1A**). Conversely, the expression of Pygo2 in GC tissues was found to be significantly elevated compared to that in normal tissues (**Figure 1B**). IHC staining of Pygo2 protein was performed on tissue sections from 30 patients with GC and normal tissues. The results demonstrated that the expression of Pygo2 in GC tissues was significantly higher than in normal tissues (**Figures 1C, D**). These results indicate that the expression of Pygo2 in GC tissues is increased, but there are certain differences between different GC patients.

### 3.2 Pygo2 is expressed in tumor cells and T cells

**Figure 2:**
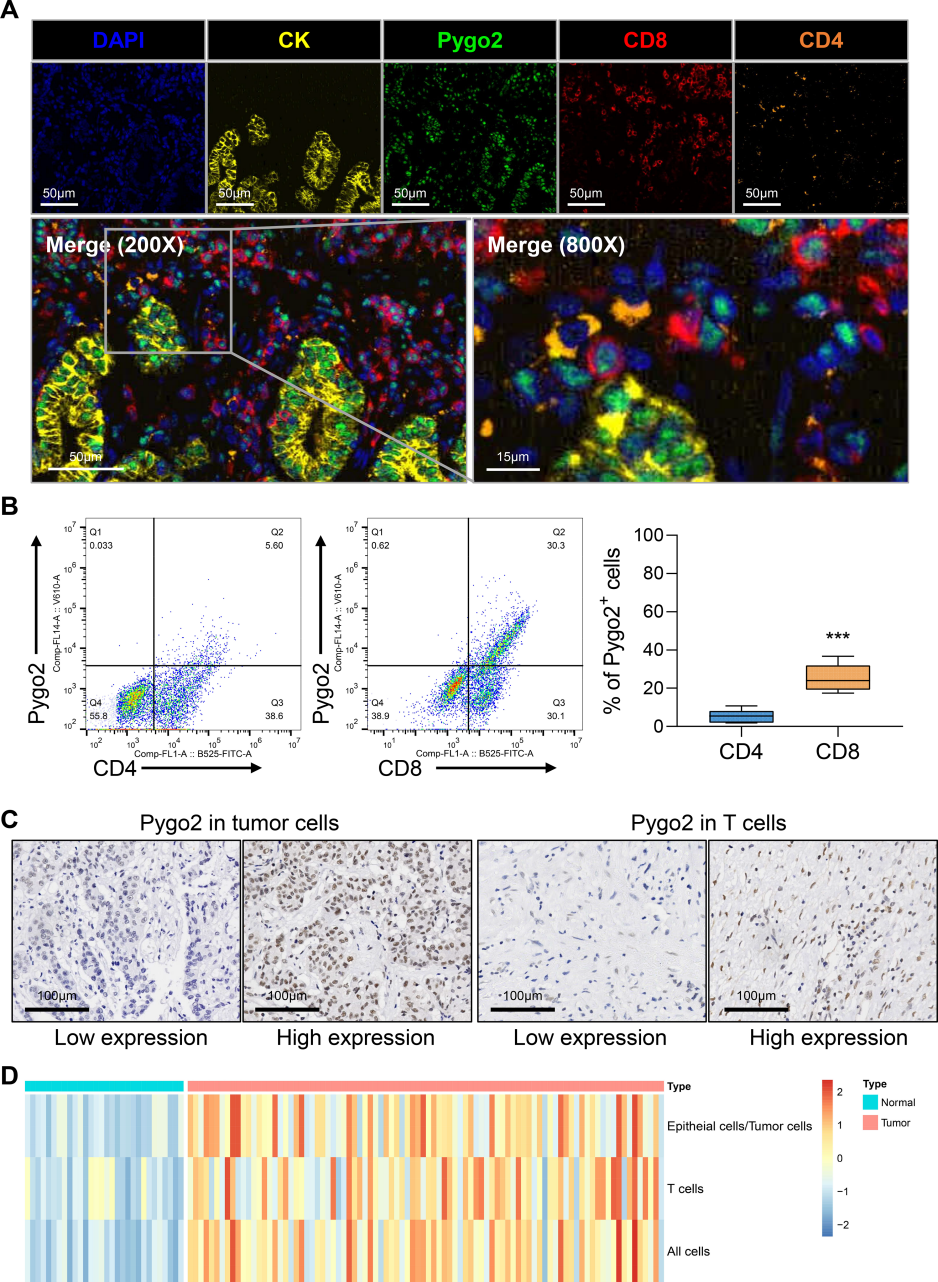
Pygo2 was expressed in tumor cells and T cells. **(A)** Representative mIHC staining showing the location of Pygo2 (green), CD8 (red) and CD4 (orange) cells in relationship to cytokeratin (CK, yellow) positive tumor islands. Nuclei are pseudocoloured blue. **(B)** Flow cytometry was used to detect the expression ratio of Pygo2 in CD4 and CD8 T cells in GC tissues. **(C)** Representative IHC images of Pygo2 revealed high and low in tumor cells and T cells, respectively. **(D)** Heat map of the expression levels of Pygo2 in tumor cells, and T cells in GC and normal tissues.

The expression of Pygo2 in GC tissue was examined using single-cell sequencing analysis. The analysis revealed that the tissue can be categorized into eight distinct cell types: T cells, epithelial cells (tumor cells), B cells, endothelial cells, macrophages, natural killer (NK) cells, fibroblasts, and mast cells (**Figure 1E**, **Supplementary Figure S1**). Further investigation indicated that Pygo2 was predominantly expressed in T cells and tumor cells (**Figure 1F**). The localization of Pygo2 in GC tissue was corroborated through mIHC staining, which demonstrated that Pygo2 was primarily found in CK cells and T cells, with a notable concentration in the cell nucleus. Pygo2 expression was predominantly observed in CD8+ T cells, whereas CD4+ T cells exhibited only minimal expression of Pygo2 (**Figure 2A**). Meanwhile, flow cytometry was used to analyze the expression of Pygo2 in T cells in fresh tumor tissues of GC patients. It was found that Pygo2 was mainly expressed on some CD8+ T cells but rarely CD4+ T cells (**Figure 2B**). Furthermore, we separately assessed the expression levels of Pygo2 in tumor cells, and T cells in GC and normal tissues (**Figure 2C**). The results showed that the expression of Pygo2 in tumor cells, and T cells were both higher in GC, compared with normal gastric tissues (**Figure 2D**). These findings suggest that Pygo2 is primarily expressed in tumor cells and CD8+ T cells within GC tissue. The nuclear concentration of Pygo2 implies that it may play specific functional roles within the cell nucleus.

### 3.3 The relationship between Pygo2 expression and clinical pathology as well as prognosis

We conducted an analysis of Pygo2 expression across various cell types within GC tissue and examined its correlation with clinical pathological parameters. The findings indicated that there were no significant clinical pathological differences in Pygo2 expression between the overall cells and tumor cells (**Table 1**). However, a correlation was observed between Pygo2 expression and the advanced T stage and N stage in T cells (**Table 1**). These findings suggest that Pygo2-positive T cells (Pygo2+ T) may serve as a potential pathological diagnostic marker for GC.

**Table 1:** Clinicopathological characteristics and staining patterns of Pygo2 in gastric cancer.

Variables	Total	Pygo2 in all cells		*P* value	Pygo2 in tumor cells		*P* value	Pygo2 in T cells		*P* value
		High	Low		High	Low		High	Low	
Age				0.808			0.225			0.627
≤ 60	168	83	85		79	89		86	82	
> 60	114	58	56		62	52		55	59	
Gender				0.626			0,808			0.144
Male	170	83	87		84	86		79	91	
Female	112	58	54		57	55		62	50	
Differentiation				0.819			0.991			0.622
Well/moderate	104	56	48		55	49		50	54	
Poor	178	85	93		86	92		91	87	
T stage				0.342			0.113			< 0.001
T1/2	48	27	21		29	19		12	36	
T3/4	234	114	120		112	122		129	105	
N stage				0.190			0.074			0.012
N0-1	137	74	63		76	61		58	79	
N2-3	145	67	78		65	80		83	62	

**Figure 3:**
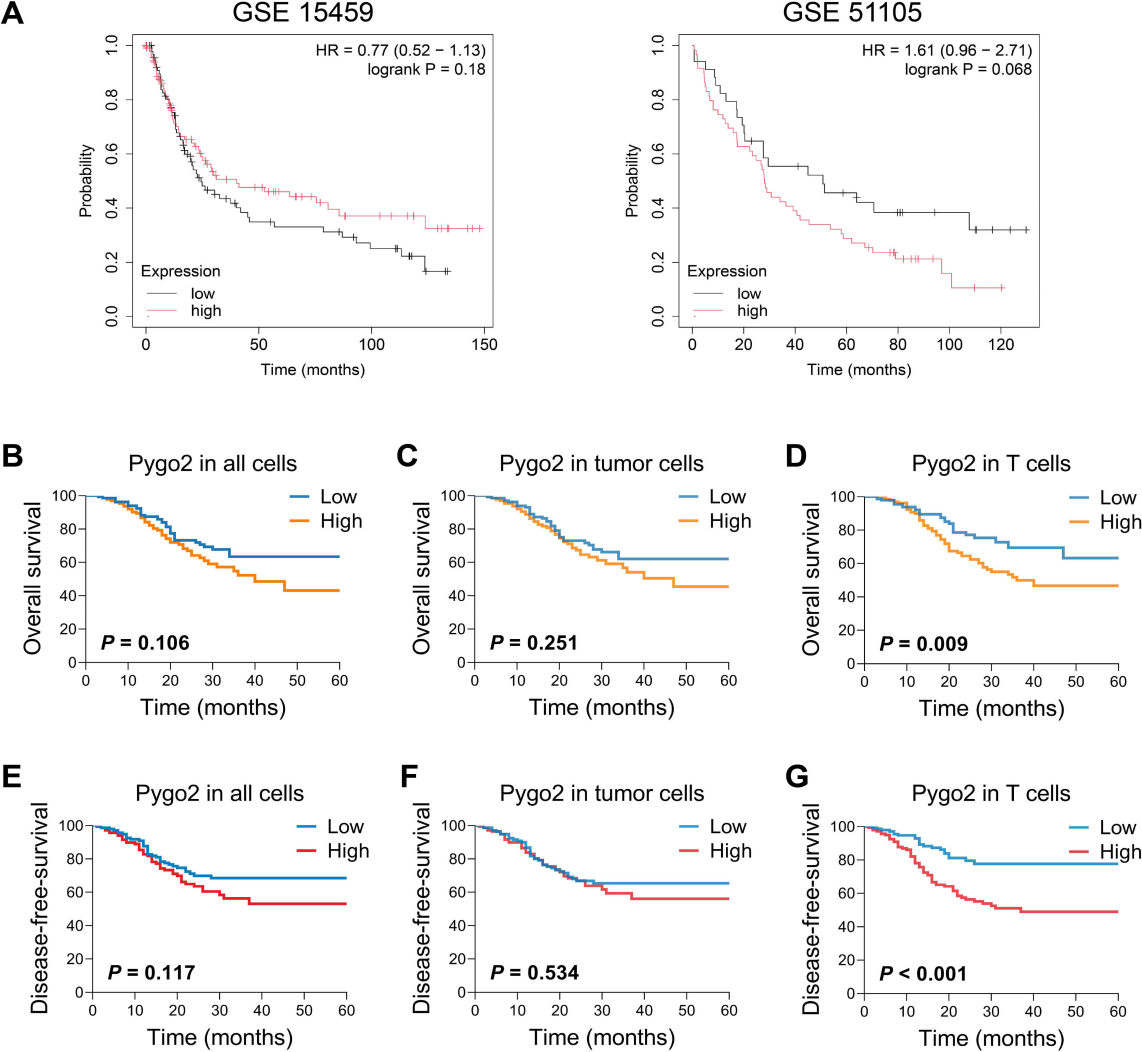
Relationship between Pygo2 expression and prognosis in patients with GC. **(A)** The relationship between Pygo2 mRNA and overall survival (OS) of GC patients was analyzed by KM Plotter analyses. **(B-D)** Our cohort analyzed the relationship between Pygo2 protein expression and OS in all cells **(B)**, tumor cells **(C)**, and T cells **(D)**, respectively. **(E-G)** Our cohort analyzed the relationship between Pygo2 protein expression and DFS in all cells **(E)**, tumor cells **(F)**, and T cells **(G)**, respectively.

To investigate the correlation between Pygo2 expression and the prognosis of GC, we analyzed the results from the GSE15459 database. The results indicated that patients with high expression of Pygo2 in GC tissues have a higher survival rate. Conversely, an analysis of the GSE51105 database produced contradictory findings (**Figure 3A**). This discrepancy suggests that the role of Pygo2 in GC prognosis may be complex and potentially influenced by the cellular localization of Pygo2 expression. Further examination demonstrated that the expression of Pygo2 in overall cells and tumor cells did not significant correlation with patient survival (**Figures 3B, C**). However, a negative correlation was observed between Pygo2 expression in T cells and patient survival rate (**Figure 3D**). Meanwhile, we analyzed the relationship between Pygo2 expression and disease-free survival (DFS). The results showed that high Pygo2+ T cell infiltration was associated with poor DFS. However, the expression of PYGO2 in tumor cells had no significant correlation with DFS (**Figures 3E–G**). These results imply that Pygo2+ T cells could serve as a potential prognostic marker for GC.

### 3.4 Correlation between Pygo2^+^ T cells and immune microenvironment

**Figure 4:**
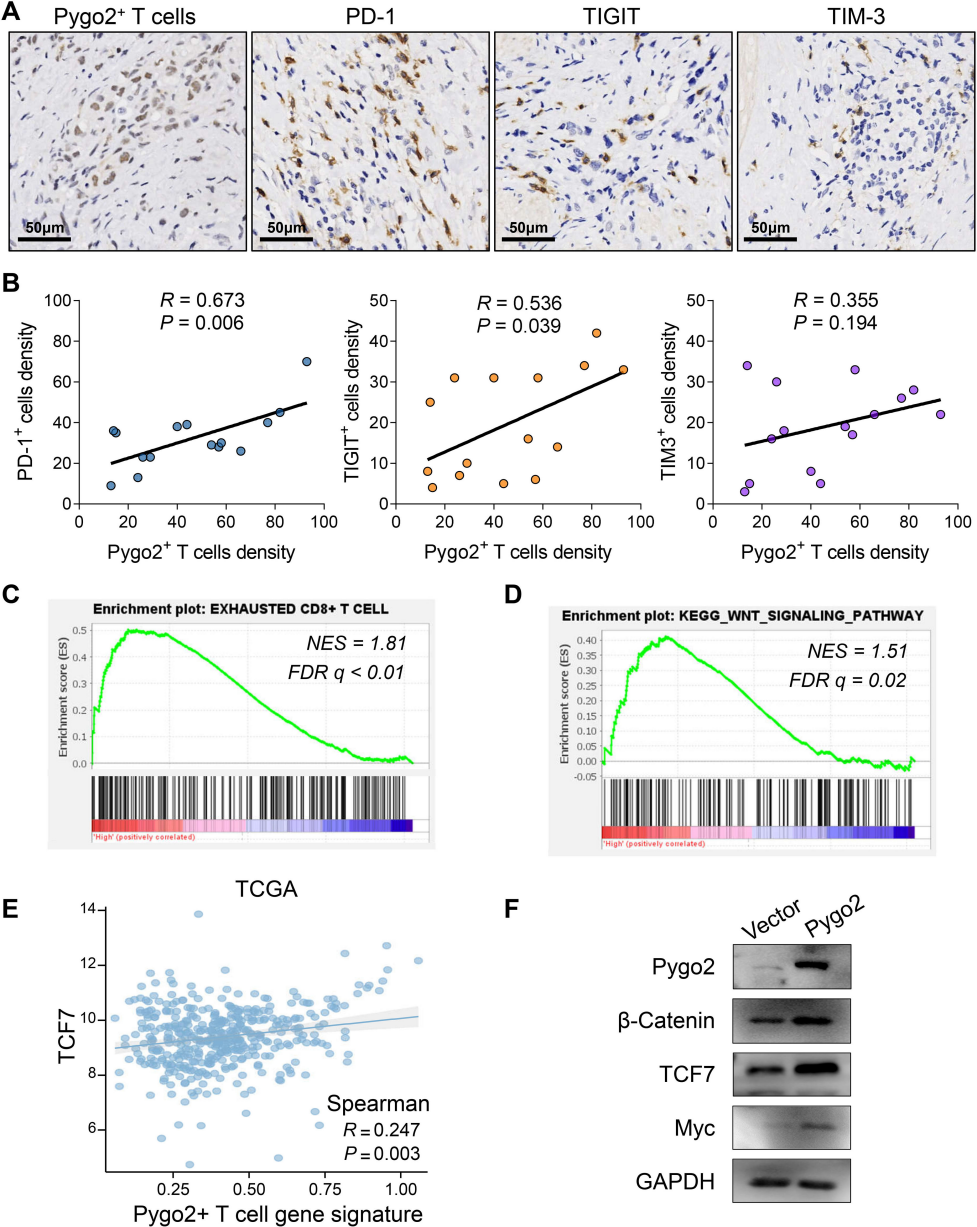
Correlation between Pygo2+ T cells and immune microenvironment. **(A)** IHC analysis of Pygo2^+^ T cells, PD-1^+^ cells, TIGIT^+^ cells, and TIM-3^+^ cells in GC. **(B)** Correlation analysis of Pygo2^+^ T cells, PD-1^+^ cells, TIGIT^+^ cells, and TIM-3+ cells. **(C, D)** GSEA of exhausted T cell related gene signature **(C)** and WNT signaling gene signature **(D)** comparing high Pygo2^+^ T cells and low Pygo2^+^ T cells gene signature group in TCGA database. **(E)** The correlation analysis between Pygo2^+^ T cells gene signature and TCF7 expression was obtained from TCGA database. **(F)** The expression of Pygo2, β-Catenin, Myc, and TCF7 was detected by Western blot after overexpression of Pygo2 in T cells.

T cells are important immune cells in tumor microenvironment. To explore the relationship between Pygo2+ T cells and immune microenvironment, we analyzed the functional differences between Pygo2+ CD8+ T cells and Pygo2- CD8+ T cells using the ssGSEA method through single-cell sequencing data. The results showed that Pygo2+ CD8+ T cells exhibited significantly enhanced apoptotic signaling, suggesting potential functional exhaustion in this subset of T cells (**Supplementary Figure S2**). Then, we investigated the relationship between Pygo2+ T cells and immune checkpoints through IHC staining (**Figure 4A**). The finding indicated that the density of Pygo2+ T cells was positively correlated with the presence of PD-1+ T cells, TIGIT+ T cells, and TIM3+ T cells (**Figure 4B**). We defined the gene set of Pygo2+ T cells using single-cell RNA sequencing analysis. Consistently, Pygo2 T cells gene signature also showed significant positive correlation with PD-1, TIGIT and TIM3 gene expression by analyzing TCGA data (**Supplementary Figure S3**). To further explore the effect of Pygo2 on immune microenvironment, GSEA results showed that this gene set was not only associated with T cell exhaustion (**Figure 4C**), but also associated with the activation of the WNT signaling pathway (**Figure 4D**). We identified a positive correlation between Pygo2+ T cell gene signature and TCF7 (**Figure 4E**). We isolated and cultured peripheral blood lymphocytes and overexpressed Pygo2 levels. Additionally, immunoimprinting showed that β-Catenin (key protein of WNT signaling), Myc (WNT target protein) and TCF7 (critical transcription factor of T cell exhaustion) increased significantly after overexpression of T cell Pygo2 (**Figure 4F**). Additionally, we analyzed the interactions between Pygo2+ CD8+ T cells and Pygo2- CD8+ T cells with tumor microenvironment cells. The results showed that Pygo2+ CD8+ T cells exhibited significantly stronger interactions with tumor cells and macrophages compared to Pygo2- CD8+ T cells (**Supplementary Figure S4**). Further analysis revealed that Pygo2+ CD8+ T cells primarily engage with tumor cells and macrophages through immune checkpoint receptor-ligand pairs, such as CD74, CD55, and SPP1 (**Supplementary Figure S5**). Collectively, these results suggest that Pygo2 is associated with the immunosuppressive microenvironment.

### 3.5 Pygo2^+^ CD8^+^ T cells predict the efficacy of immune checkpoint inhibitor treatment in GC

**Figure 5:**
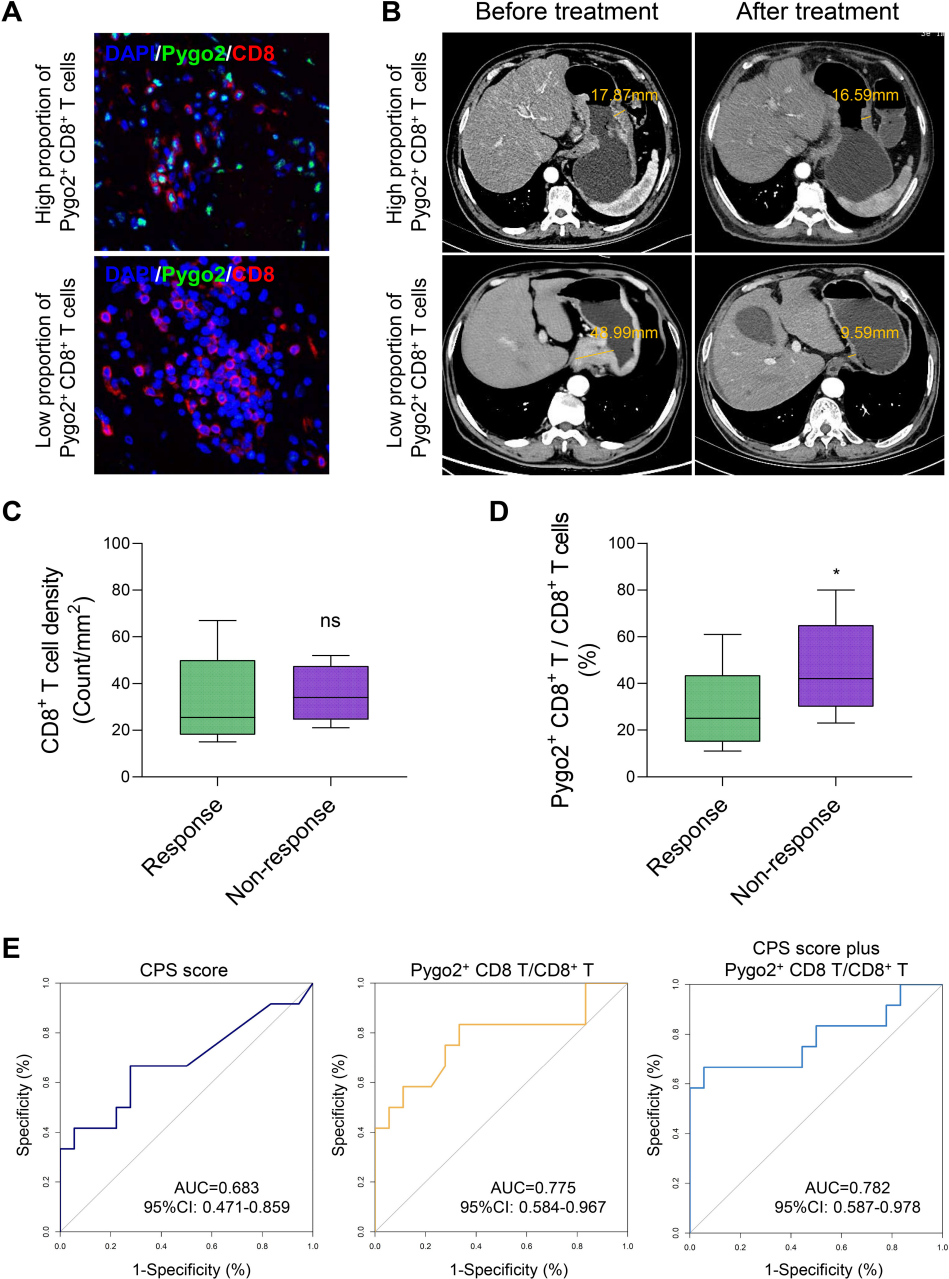
The percentage of Pygo2^+^ CD8^+^ T can determine the effectiveness of neoadjuvant immunotherapy. **(A)** Representative images of IHC staining for Pygo2 and CD8 T cells. **(B)** Corresponding CT images revealing the correlation between the infiltration Pygo2^+^ CD8^+^ T cells and response to immunotherapy. **(C)** CD8^+^ T cell infiltration levels in patients who responded and did not respond to immune checkpoint inhibitor (ICI) therapy. **(D)** The percentage of Pygo2^+^ CD8^+^ T cells to CD8^+^ T cells in patients who responded and did not respond to ICI therapy. **(E)** ROC curve of CPS score, percentage of Pygo2^+^ CD8^+^ T cells, and CPS score plus percentage of Pygo2^+^ CD8^+^ T cells.

Currently, challenges exist in the immunotherapy of GC, including limited treatment efficacy and the difficulty in effectively identifying appropriate patient populations (21, 22). These issues are closely linked to the absence of specific biomarkers for GC. In light of this, we utilized gastroscopic tissues from GC patients who had not been treated with ICI for mIHC staining, aiming to categorize patients based on varying ratios of Pygo2^+^ CD8^+^ T cells, and then screened out GC patients with different Pygo2^+^ CD8^+^ T cell ratios (**Figure 5A**). Following ICI treatment, the changes in GC tissues of patients were observed by CT images (**Figure 5B**). No correlation was observed between the overall density of CD8^+^ T cells and ICI therapy (**Figure 5C**). Conversely, the findings showed a negative correlation between the ratio of Pygo2^+^ CD8^+^ T cells density to total CD8^+^ T cells density and the efficacy of ICI treatment (**Figure 5D**). To assess the predictive value of Pygo2^+^ T cells in comparison to the CPS score, we constructed a receiver operating characteristic (ROC) curve (**Figure 5E**). The analysis revealed that the area under the curve (AUC) for percentage of Pygo2^+^ CD8^+^ T cells (AUC=0.775, 95%CI 0.584-0.967) was superior to that of the CPS score (AUC=0.683, 95%CI 0.471-0.859), suggesting that the infiltration level of Pygo2^+^ T cells possessed significant predictive power for evaluating the responsiveness of patients to immunotherapy. The above results show that Pygo2^+^ CD8^+^ T cells may serve as a potential biomarker for predicting the effectiveness of ICI treatment in GC patients.

## 4 Discussion

Pygo2 plays a significant role in various malignancies, including prostate cancer, breast cancer, and esophageal cancer (13, 18, 19, 23). However, there is a paucity of studies investigating the role and underlying mechanisms of Pygo2 in GC. In light of this, the present study aimed to preliminarily examine the functional mechanisms of Pygo2 in GC tissue. The findings revealed that the expression of Pygo2 was markedly elevated in GC tissue, with heightened expression predominantly observed in tumor cells and CD8^+^ T cells. Furthermore, this study found for the first time that Pygo2^+^ T cells are significantly associated with exhausted T cells, thereby influencing the immunosuppressive microenvironment of GC. Through pathological investigations and prognostic analyses, we established that Pygo2^+^ T cells possess the potential to serve as a prognostic biomarker for the pathological diagnosis and immunotherapy of GC.

Previous research on the targets of Pygo2 has predominantly concentrated on tumor cells (13, 24–26). The expression of Pygo2 on T cells has not been reported. T cells exert cytotoxic effects on neoplastic cells (27–29). They serve as a primary component of the immune response against tumors by recognizing and eliminating malignant cells. In prostate cancer, it was observed that the expression of Pygo2 in cancer cells was negatively correlated with the infiltration of T cells. These findings indicate that Pygo2 may play a role in tumor immune evasion. However, there is a paucity of studies examining the relationship between Pygo2 expression and T cells. Consequently, our study aimed to investigate the association between Pygo2 expression and T cells in GC. Notably, we identified a novel T cell sub-type, Pygo2^+^ T cells. Within the cancer microenvironment, tumor cells can induce T cell exhaustion through various mechanisms, thereby evading immune surveillance and attack (13, 30, 31). Our findings indicated that Pygo2^+^ T cells might be linked to exhausted T cells, thereby affecting the immunosuppressive microenvironment within the GC. This discovery not only elucidates the tripartite nexus among Pygo2 expression, T cell exhaustion, and tumorigenesis but also offers a new biological target for cancer immunotherapy.

Previous research regarding the role of Pygo2 in tumor prognosis has predominantly concentrated on the analysis of tumor tissues or cellular models. For instance, in the context of brain gliomas, the expression levels of Pygo2 have been found to correlate with the extent of tumor progression. This research suggests that Pygo2 may be instrumental in the initiation and advancement of brain gliomas (26). However, the prognostic significance of Pygo2 in certain tumors appears to be relatively limited (24). Our investigation revealed that the majority of GC patients exhibited Pygo2 expression in their tumor cells, which might contribute to the restricted prognostic impact of Pygo2. Furthermore, this study identified that the role of Pygo2 in GC prognosis was contentious, potentially due to the cell localization of Pygo2 expression. Additional analyses indicated that Pygo2 expression in tumor cells did not demonstrate a significant correlation with patient survival. Conversely, Pygo2 expression in T cells was found to be negatively correlated with survival outcomes. Pathological findings further corroborated this perspective. Consequently, Pygo2^+^ T cells may possess potential prognostic value in the context of immunotherapy for GC.

We found that high expression of Pygo2 in T cells was associated with poor patient prognosis. Multiplex immunofluorescence staining revealed that Pygo2 was primarily expressed in CD8^+^ T cells rather than CD4^+^ T cells. CD8^+^ T cells are the most critical anti-tumor immune T cells, but tumor-infiltrating T cells undergo exhaustion due to immune checkpoint mechanisms. Therefore, we hypothesized that Pygo2 expression in T cells might be linked to T cell exhaustion. We investigated the relationship between Pygo2+ T cells and immune checkpoints through IHC staining. The finding indicated that the density of Pygo2^+^ T cells was positively correlated with the presence of PD-1^+^ T cells, TIGIT^+^ T cells, and TIM3^+^ T cells. Moreover, GSEA results showed that Pygo2 was not only associated with T cell exhaustion, but also associated with the activation of the WNT signaling pathway. Pygo2 is a recently identified component of the Wnt signaling pathway. It activates this pathway by promoting the accumulation of β-catenin and facilitating its translocation into the nucleus. This translocation enhances the transcriptional activity of TCF/LEF family transcription factors (15, 32). Our study demonstrated a positive correlation between Pygo2 and TCF7. Previous research has established that TCF7 is a critical transcription factor associated with T cell exhaustion and it predominantly express in early exhausted T cells (33–35). This result suggests that Pygo2 is associated with the immunosuppressive microenvironment. The expression of T cell exhaustion related markers, such as PD-1 and CTLA4, is markedly high prior to or during the early phases of treatment, which may enhance the predictive capacity for the clinical outcomes of immunotherapy (36–38). Consequently, Pygo2 holds promise as a potential biomarker for forecasting the efficacy of immunotherapy in GC. Furthermore, our study revealed that the density of Pygo2^+^ T cells was positively correlated with the number of PD-1^+^ T cells, TIGIT^+^ T cells, and TIM3^+^ T cells. This observation further substantiates the notion that Pygo2 may serve as a valuable marker for predicting the effectiveness of immunotherapy in GC.

## 5 Conclusions

In summary, the results of the present cohort study highlight Pygo2^+^ T cells as predictors of poorer prognostic outcomes in patients with GC. The dense infiltration of Pygo2^+^ T cells is associated with the immunosuppressive microenvironment, resulting in a decrease in the effectiveness of immunotherapy. Consequently, Pygo2^+^ T cells may serve as a potential biomarker of tumor immunotherapy efficacy. Further studies are essential to explore therapeutic targeting Pygo2^+^ T cells.

## Data Availability

The datasets presented in this study can be found in online repositories. The names of the repository/repositories and accession number(s) can be found in the article/**Supplementary Material**.

## Supplementary material

Supplementary Figure 1 Cell markers used to identify cell types in single cell sequencing analysis.

Supplementary Figure 2 The functional enrichment in Pygo2^+^ CD8^+^ T cells through ssGSVA analysis.

Supplementary Figure 3 Correlation analysis of Pygo2^+^ T cells gene signature, PD-1, TIGIT, and TIM-3 expression from TCGA database.

Supplementary Figure 4 Interactions between Pygo2^+^ CD8^+^ T cells and Pygo2^-^ CD8^+^ T cells with tumor microenvironment cells.

Supplementary Figure 5 The receptor-ligand networks underlying Pygo2^+^ CD8^+^ T cell communication with TME populations were systematically analyzed.
