# Supplementary figures and images for: Pygo2+ T cells possess immunosuppressive features and inferior immunotherapeutic response in gastric cancer
Source: Front Immunol. 2025 Jul 23;16:1596434. doi: 10.3389/fimmu.2025.1596434 (PMC12326480; doi:10.3389/fimmu.2025.1596434)

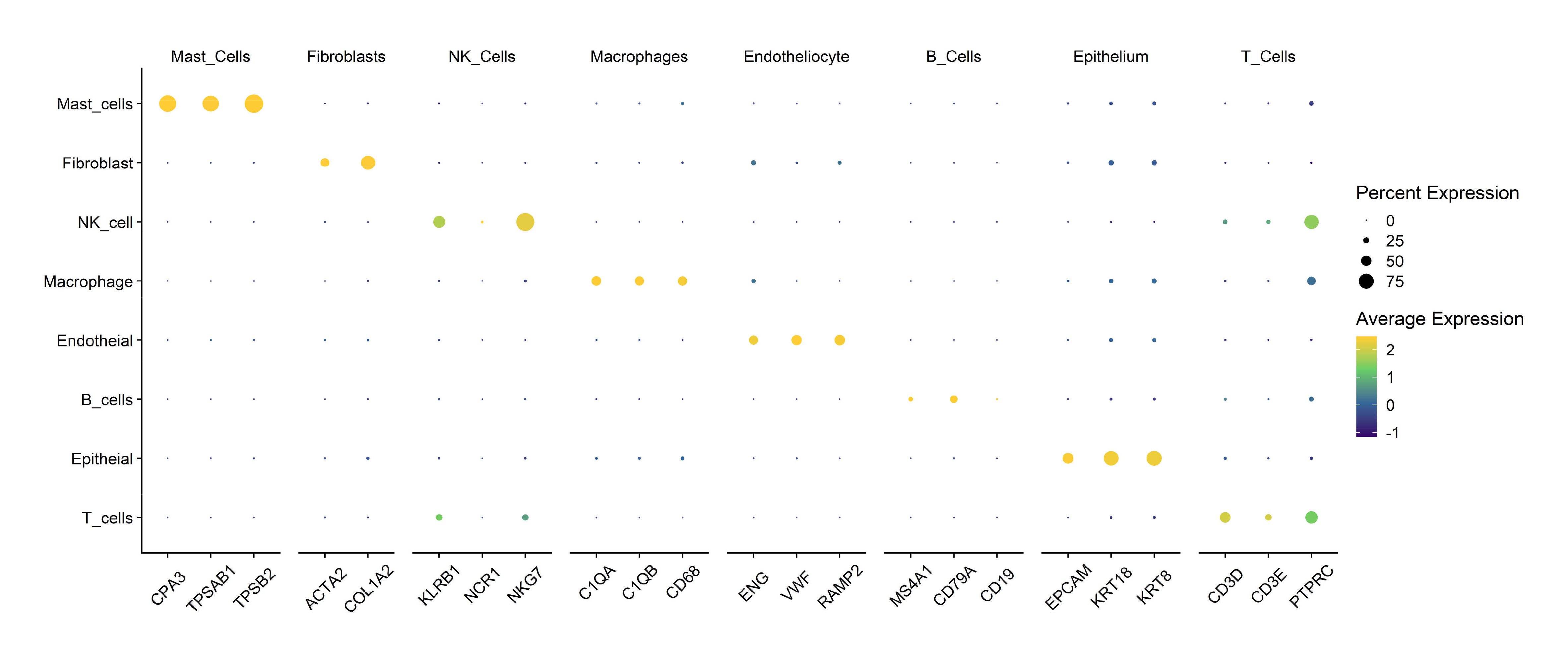

Supplement: Supplementary Figure 1 — Cell markers used to identify cell types in single cell sequencing analysis. [file Image1.jpeg]

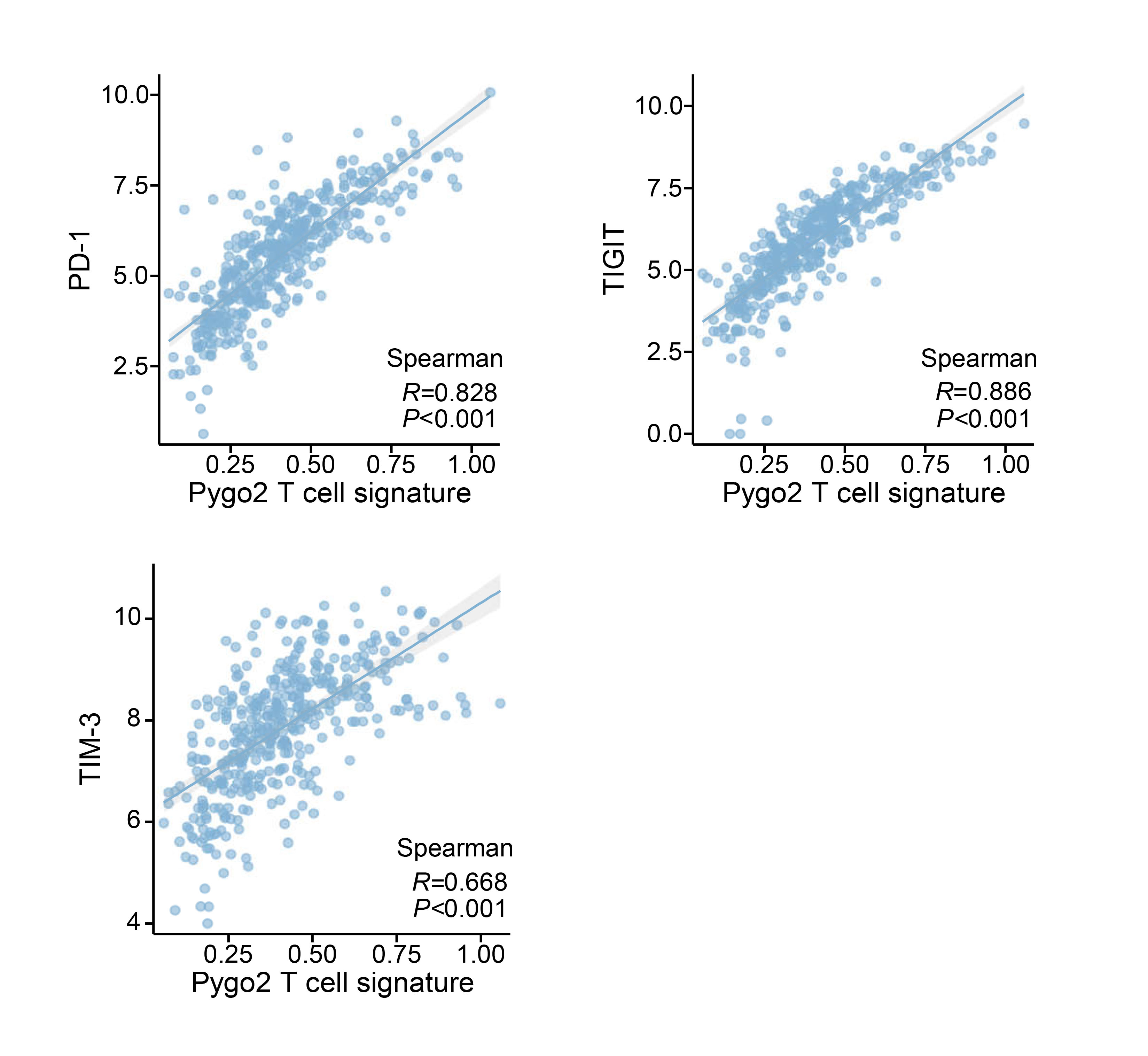

Supplement: Supplementary Figure 3 — Correlation analysis of Pygo2+ T cells gene signature, PD-1, TIGIT, and TIM-3 expression from TCGA database. [file Image3.jpeg]

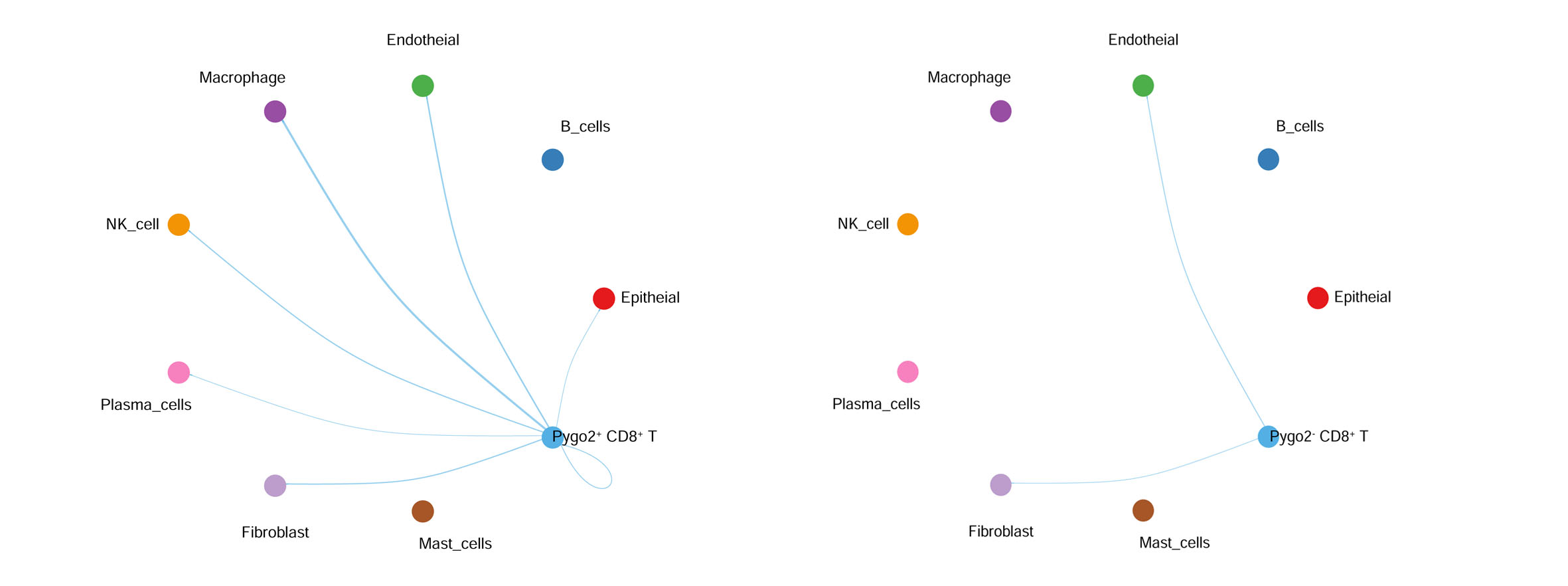

Supplement: Supplementary Figure 4 — Interactions between Pygo2+ CD8+ T cells and Pygo2- CD8+ T cells with tumor microenvironment cells. [file Image4.jpeg]

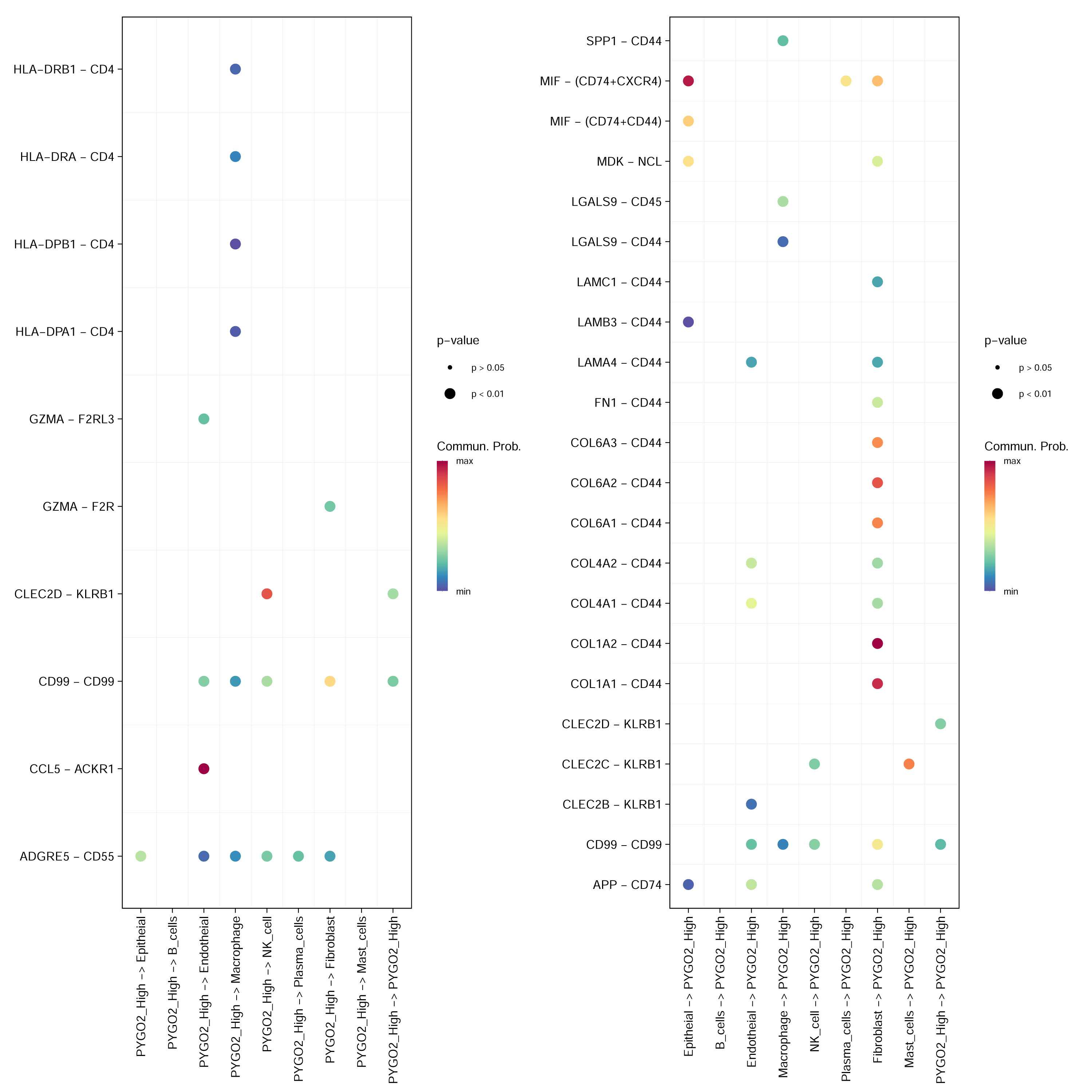

Supplement: Supplementary Figure 5 — The receptor-ligand networks underlying Pygo2+ CD8+ T cell communication with TME populations were systematically analyzed. [file Image5.jpeg]
