# Supplementary material for: Pygo2+ T cells possess immunosuppressive features and inferior immunotherapeutic response in gastric cancer
Source: Front Immunol. 2025 Jul 23;16:1596434. doi: 10.3389/fimmu.2025.1596434 (PMC12326480; doi:10.3389/fimmu.2025.1596434)
Supplement: Supplementary file 6 [file DataSheet1.pdf]

Table S1. Information on gastric cancer patients receiving neoadjuvant immunotherapy

ID	Treatment	Outcome	Age	Sex	cTNM	ypTNM
1	Sintilimab+Oxaliplatin+ Leucovorin Calcium+ 5-FU	PD	76	male	T4N3M0	T4N3M0
2	Sintilimab+Oxaliplatin+S-1	CR	55	male	T4N1M0	T1N1M0
3	Camrelizumab+Oxaliplatin+Capecitabine	SD	54	male	T3N1M0	T3N1M0
4	Sintilimab+Oxaliplatin+Capecitabine	SD	63	male	T3N2M0	T3N1M0
5	Sintilimab+Oxaliplatin+S-1	PR	57	female	T4N3M0	T3N0M0
6	Sintilimab+Oxaliplatin+S-1	CR	66	female	T4N1M0	T1N1M0
7	Sintilimab+Oxaliplatin+S-1	CR	63	male	T3N2M0	TxN0M0
8	Sintilimab+Oxaliplatin+S-1	PD	78	male	T3N2M0	T4N2M0
9	Sintilimab+Oxaliplatin+S-1	SD	67	male	T4N1M0	T3N1M0
10	Tislelizumab+Oxaliplatin+S-1	CR	65	female	T4N2M0	T2N1M0

CR: Complete Response; PR: Partial Response; SD: Stable Disease; PD: Progressive Disease; Tx: undetected cancer cell
